# Elucidating the Inhibitory Potential of Designed Peptides Against Amyloid Fibrillation and Amyloid Associated Cytotoxicity

**DOI:** 10.3389/fchem.2018.00311

**Published:** 2018-08-03

**Authors:** Mohammad K. Siddiqi, Parvez Alam, Tabish Iqbal, Nabeela Majid, Sadia Malik, Saima Nusrat, Aftab Alam, Mohd R. Ajmal, Vladimir N. Uversky, Rizwan H. Khan

**Affiliations:** ^1^Interdisciplinary Biotechnology Unit, Aligarh Muslim University, Aligarh, India; ^2^Center for Interdisciplinary Research in Basic Science, Jamia Millia Islamia, New Delhi, India; ^3^Protein Research Group, Institute for Biological Instrumentation of the Russian Academy of Sciences, Moscow, Russia; ^4^Department of Biological Sciences, Faculty of Sciences, King Abdulaziz University, Jeddah, Saudi Arabia; ^5^Department of Molecular Medicine and USF Health Byrd Alzheimer's Research Institute, Morsani College of Medicine, University of South Florida, Tampa, FL, United States

**Keywords:** amyloid, inhibitor, thioflavin T assay, hemolysis, TEM, human insulin

## Abstract

Inhibition of fibrillation process and disaggregation of mature fibrils using small peptide are the promising remedial strategies to combat neurodegenerative diseases. However, designing peptide-based drugs to target β-sheet-rich amyloid has been a major challenge. The current work describes, for the first time, the amyloid inhibitory potential of the two short peptides (selected on the basis of predisposition of their amino acid residues toward β-sheet formation) using combination of biophysical, imaging methods, and docking approaches. Results showed that peptides employed different mechanisms to inhibit the amyloid fibrillation. Furthermore, they were also effective in blocking the amyloid fibrillation pathway. In contrary to the insulin fibrillar mesh, significantly less fibrillar species appeared in the presence of peptides, as confirmed by transmission electron microscopy. Circular dichroism analysis indicated that although peptides did not stabilize the native state of insulin, they inhibited amyloid aggregation by reducing the formation of β-sheet rich structures. Hemolytic assay revealed the non-hemolytic nature of the species formed when insulin was co-incubated with the peptides. Therefore, despite the inherent potential to form β-sheet structure, these peptides inhibited the amyloid formation and potentially can be used as therapeutics for the treatment of amyloid-related diseases.

## Introduction

Aggregation and disaggregation of proteins and peptides are at the center of interest in various scientific fields, spanning from nanosciences, membrane technology, gelation, drug delivery to drug discovery, and medicine. Excessive deposition of stable, ordered, or amorphous protein aggregates in organs and tissues leads to crucial biological dysfunctions and deleterious pathological consequences associated with amyloidosis. Amyloidoses, such as Alzheimer's disease (AD), Parkinson's disease (PD), Huntington's disease (HD), amyotrophic lateral sclerosis (ALS), frontotemporal dementia (FTD), diabetes type II, a set of prion disorders, and many others, are characterized by the presence of amyloid fibrils, the unusually stable aggregates of originally soluble proteins and peptides (Sipe and Cohen, 2000; Lashuel et al., 2002; Chiti and Dobson, 2006; Chaturvedi et al., 2016). Amyloidosis is often initiated at biomembranes, and the attachment of aggregated proteins to the cell membranes may result in membrane permeabilization that ultimately causes cell dysfunction and apoptosis. In addition to proteins associated with amyloid diseases, non-pathological proteins also form amyloid fibrils with characteristics, such as fibrillar morphology, biochemical properties, similar to those of the amyloidogenic proteins. This suggests the inherent property of polypeptides to form amyloid fibrils (Chiti et al., 2003; Chiti and Dobson, 2006; Siddiqi et al., 2016). In view of such important observations and disease implications, considerable effort has been devoted in understanding protein fibrillation process and to finding the compounds/small molecules that have the ability to interfere with the fibrillation process. Despite intensive research, the exact mechanism for amyloid fibrillation has yet to be fully understood.

Human insulin (HI) is a 51-amino acid residue-long hypoglycemic hormone that undergoes fibrillation. For example, being a crucial drug for treating diabetes, insulin is associated with the injection amyloidosis, found at the site of recurrent insulin injections (Okamura et al., 2013). Furthermore, fibril formation by insulin has been a trouble for the long-distance transportation and long-term storage, thereby causing the reduced effectiveness of this important drug. Therefore, insulin is an exceptional model protein to study protein fibrillogenesis.

Insulin is a covalent dimer of two polypeptide chains (chain A and chain B) having 21 and 30 amino acid residues, respectively. Both the chains are crossed linked through disulfide bonds. In its native state, insulin principally exists in α-helical structure, where residues A1–A8 and A13–A20 form two α-helices in chain A, whereas residues B11–B19 form an α-helix in chain B. Furthermore, insulin may adopt different oligomeric conformations, such as dimer, tetramer, and hexamer, depending on the solution condition (Nettleton et al., 2000; Hua and Weiss, 2004). At physiological pH, insulin exists in hexameric form whereas at low pH it exists as either monomeric or dimeric state which is more prone to form amyloid.

*In vitro* insulin amyloid fibrils are formed under certain destabilizing environments, such as high temperature, increased ionic strength, exposure to hydrophobic surface, low pH, and shaking (Jansen et al., 2005; Jayamani and Shanmugam, 2014; Kitagawa et al., 2015). It is still under argument that either an intermolecular hydrophobic force or electrostatic attraction is responsible for the amyloidogenesis. It is commonly accepted that fibrillogenesis is commenced due to the partial unfolding of insulin monomers, followed by their association to form protein oligomers and ß-sheet rich amyloid aggregates (Bekard and Dunstan, 2009; Librizzi et al., 2014). Consequently, understanding of the molecular basis of insulin fibrillation could be of great assessment in the direction of modeling the amyloidogenic pathways and toward developing delivery systems in the treatment of amyloid associated diseases.

Several potent compounds have been reported to have the ability of amyloid inhibition (Awasthi et al., 2016; Mohamed et al., 2016; Nusrat et al., 2016), which include such as polyphenols, nanoparticles, flavonoids, vitamins, various drugs (Lee et al., 2012; Alam P. et al., 2016; Nedumpully-Govindan et al., 2016; Nusrat et al., 2017; Usmani et al., 2017). Certainly, detecting and uncovering the mechanisms of amyloid aggregation with an insight at molecular level are of diagnostic significance and have therapeutic consequences. In this study, we have chosen to test the effect of two designed peptides P4 (Val-Ile-Phe-Tyr-Thr) and P5 (Val-Val-Val-Val-Val) (Supplementary Figure S1). These peptides were selected on the basis of the predisposition of their amino acid residues to acquire a particular secondary structure (based on their frequencies of being found in particular types of secondary structure). For example, the predispositions to form random coil structure for Val, Ile, Phe, Tyr, and Thr residues are 0.47, 0.51, 0.58, 1.05, and 1.03; for α-helix they are 0.91, 0.97, 1.07, 0.72, and 0.82; and for β-sheet structure are 1.49, 1.45, 1.32, 1.25, and 1.21, respectively (Berg et al., 2002). Although P5 and P4 both possess the predisposition to form the β-sheet structure, P5 consists of residues having the highest β-structure forming frequency (1.49), whereas P4 is made up of residue with mixture of frequencies (1.49, 1.45, 1.32, 1.25, and 1.21).

Herein, we have delineated the anti-amyloidogenic, fibril disaggregating, as well as cell protective effects of P4 and P5 against the insulin amyloid fibrillation by employing biophysical, microscopic, cell cytotoxicity, and computational studies. The anti-aggregation action of P4 and P5 against insulin fibrillation may possibly deliver an enhanced vision of structurally similar compounds against the aggregation of protein and might prove useful in designing suitable inhibitors which may act against amyloid associated disorders.

## Experimental procedures

### Chemical and buffers

Human insulin (HI), Thioflavin T (ThT), and 1-anilino 8 naphthalene sulfate (ANS) were purchased from Sigma Aldrich, India. Two short peptides viz: VVVVV and VITYF were purchased from GenScript (Piscataway, NJ). The experiments were performed in 50 mM acetate buffer, pH 2.0. All other reagents used were of analytical grade.

### Sample preparation

Stock solution of insulin was prepared by dissolving insulin lyophilized powder in 50 mM acetate buffer, pH 2.0, and insulin concentration was determined by measuring absorbance at 276 nm, with the extinction coefficients of 1.0 M^−1^ cm^−1^ for 1 mg/ml. Fibrils were prepared by incubating insulin solution at 60°C for 72 h.

### ThT fluorescence measurements

ThT stock solution was prepared by dissolving ThT in double distilled water, and the resulting solution was passed through a 0.2 micron millipore filter. The concentration of ThT was determined by measuring absorbance at 412 nm with a molar extinction coefficient of 36,600 M^−1^cm^−1^. ThT fluorescence assay was carried out on Shimadzu fluorescence spectrophotometer (RF-5301 PC). Insulin samples were incubated with or without peptides; from each set, samples were withdrawn at definite time intervals and mixed with ThT to have the final protein and dye concentration of 20 μM. The ThT was excited at 440 nm, and fluorescence spectra were recorded from 460 to 650 nm. Buffer (pH 2.0) was used for dilution, and spectra were corrected for the respective blanks. All measurements were performed in triplicates. All curves were fitted as described previously (Chaturvedi et al., 2015).

### Acrylamide quenching

Acrylamide quenching fluorescence assay was carried out on Shimadzu fluorescence spectrophotometer (RF-5301 PC). Insulin samples incubated (at 60°C for 72 h) with or without peptides were mixed with acrylamide to reach a final acrylamide concentration varying from 0 to 25 mM, and the fluorescence was measured immediately. Intrinsic tyrosine fluorescence quenching was measured by exciting the protein fluorescence at 276 nm and emission spectra were recorded between 280 and 500 nm. Experiments were carried out at 25°C, and spectra were corrected for respective blanks. All measurements were performed in triplicates.

### ANS fluorescence measurements

ANS stock solution was prepared by dissolving ANS in double distilled water, and the resulting solution was passed through a 0.2 micron millipore filter. The ANS concentration was determined spectrophotometrically using the extinction coefficient of ε_350nm_ = 5,000 M^−1^cm^−1^. Insulin samples (20 μM) in absence and presence of peptides were mixed with 20-fold molar excess of ANS, and then the mixtures were incubated at room temperature in dark for 30 min. ANS fluorescence intensities were recorded with excitation at 380 nm and emission between 400 and 600 nm using a fluorescence spectrophotometer (RF-5301 PC). Excitation and emission slit widths were set at 3 and 5 nm, respectively. All measurements were performed in triplicates.

### Far-UV circular dichroism measurements

The far-UV circular dichroism (CD) spectra were recorded at 25°C on a JASCO spectropolarimeter (J-815) with a thermostatically controlled cell holder attached to a Peltier unit with the MultiTech water circulator. The experiments were carried out for insulin solutions in the absence and presence of peptides. Samples were scanned in the range of 200–250 nm in a cuvette with the path length of 0.1 cm using the scanning speed of 100 nm/min. The spectropolarimeter was thoroughly purged with nitrogen gas before starting the experiments and continuously purged till the end of experiment. All measurements were performed in triplicates. Each spectrum was an average of three scans.

### Dynamic light scattering (DLS) measurements

The changes in the aggregation behavior of insulin in the presence of different concentration of peptides were determined using dynamic light scattering (DLS). DLS measurements were conducted on a Zetasizer instrument (Nano ZS; Malvern Instruments) using disposable cuvette to determine the hydrodynamic radii (R_h_) of protein under various conditions. A 633 nm wavelength He-Ne-laser was used to detect backscattered light at a fixed angle of 173°. The cell holder was maintained at 25°C for the measurement. Scattering data were collected as an average of 3 measurements with 13 scans for each measurement. Data were processed with the Malvern Zetasizer Software (Malvern Instruments). All measurements were performed in triplicates.

### Transmission electron microscopy (TEM)

TEM images were taken on a Philips CM-10 transmission electron microscope operated at an accelerating voltage of 200 kV. The amyloid fibrillogenesis was assessed by applying 6 μL of insulin samples in the absence and presence of peptides on 200-mesh copper grid covered by carbon-stabilized formvar film. Excess of fluid was removed after 2 min, and the grids were then negatively stained with 2% (w/v) uranyl acetate. Images were viewed at 10,000 × magnification.

### Hemolytic assay

Fresh human blood was centrifuged at 1,000 g for 10 min and cell pellets were washed three times with isotonic phosphate buffer saline (PBS, pH7.4). Insulin samples with and without peptides were incubated at 60°C and the fibril formation was confirmed by ThT assay. For hemolytic assay, the cell suspension was mixed with the incubated samples and further incubated at 37°C for 60 min. An aliquot of the sample was removed and centrifuged at 1,000 g for 10 min. Erythrocyte lysis was measured by measuring absorbance of supernatant at 540 nm. The hemolytic rate was calculated with respect to the hemolysis of erythrocyte in 1% Triton X-100, which was taken as 100%. Erythrocytes treated with isotonic PBS were used as control. All measurements were performed in triplicates.

### Molecular docking study of insulin-peptide interaction

The 3D structure of human insulin (1GUJ) was downloaded from Protein Data Bank (PDB) (http://www.rcsb.org/pdb/home/home.do). From crystal structure of human insulin, the crystallographic water molecules were removed, the missing hydrogen atoms were added, and the energy level was minimized using the Swiss_PDB viewer tool (Johansson et al., 2012). The structures of both peptides were drawn in Pepdraw (http://pepdraw.com/) and converted to their 3D form. The geometry of the compounds was optimized in ChemBio3DUltra12 (PerkinElmer Informatics, Waltham, MA, USA). Finally the resulting peptide structures were saved in PDB format for further docking studies (Alam A. et al., 2016).

Docking studies yielded crucial information concerning the orientation of the inhibitors in the binding pocket of the target proteins. The docking studies were performed using Vina Autodock4.0 (Morris et al., 2009). The docking interactions were visualized with PYMOL molecular graphics system, version 1.7.4.4 (Schrödinger, LLC, and Portland, OR, USA).

### Statistical analysis

All data were presented as mean ± standard deviation from 3 independent determinations. The statistical analysis was made by performing one-way ANOVA for 3 independent determinations. Significance of results was determined as *p* ≤ 0.01, unless otherwise stated.

## Results and discussion

### Effect of short peptides on insulin fibrillation: ThT assay

The inhibitory effects of peptides (P4 and P5) on insulin fibrillation were monitored by ThT fluorescence assay. ThT fluorescence assay has been widely used to detect the formation of amyloid fibrils, because the binding of ThT to amyloid fibrils enables reduction of self-quenching by restricting the rotation of the benzothiozole and benzaminic rings, leading to a significant increase in fluorescence quantum yield. The kinetic profile of the changes in ThT fluorescence of insulin solutions is characterized by sigmoidal curve with a short lag-phase in which nucleus evolves, a growth-phase, during which fibril elongates, and, finally a plateau-phase.

**Figure 1:**
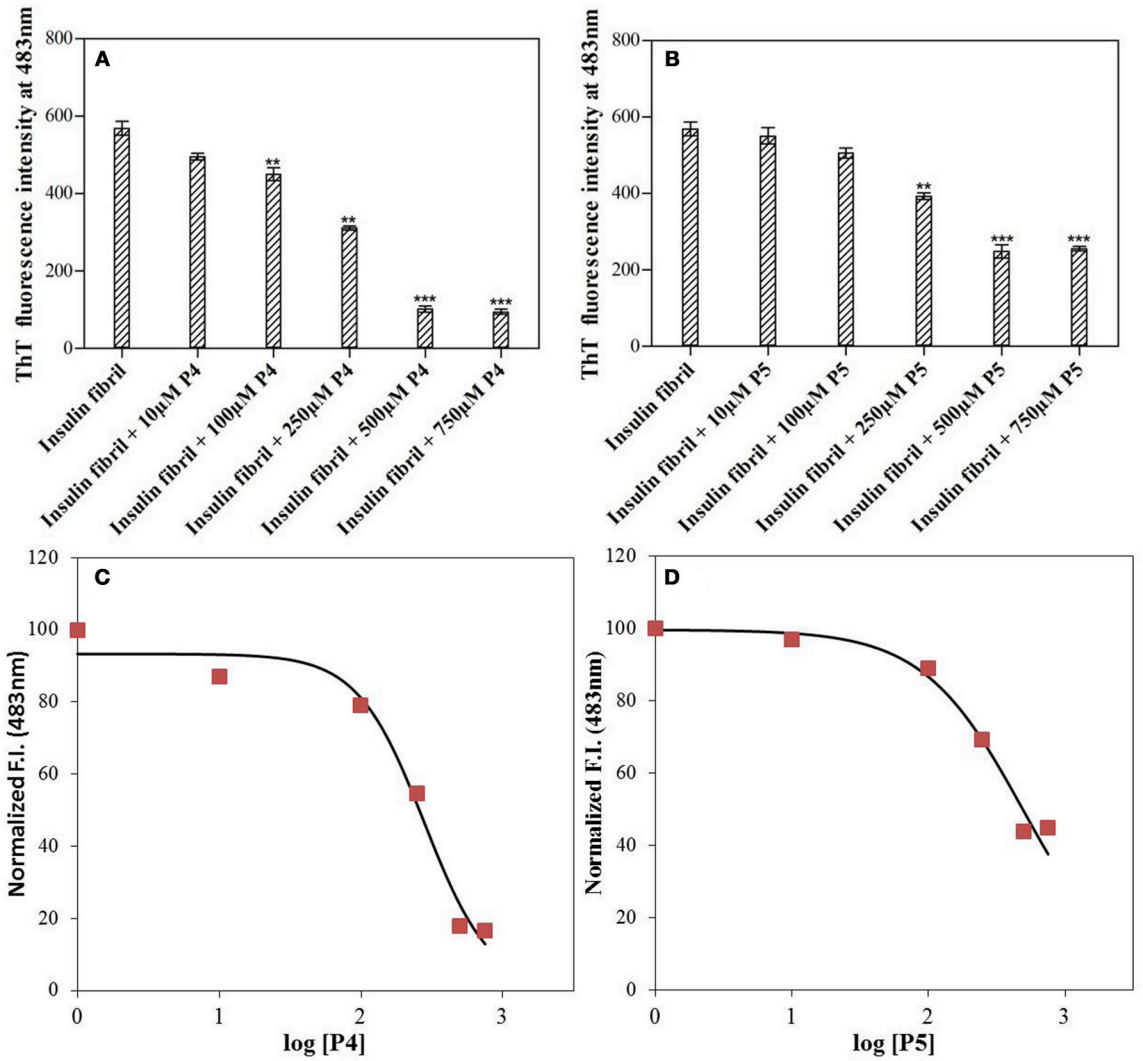
Short peptides inhibit the amyloid fibril formation by insulin. ThT fluorescence intensity at 483 nm measured for insulin solution with varying concentrations (0–750 μM) of P4 **(A)** and P5 **(B)**. ^**^P < 0.01 and ^***^P < 0.001 compared with insulin fibril. A 3-parameter dose response fitted curve for estimating IC_50_ of P4 **(C)** and P5 **(D)** against insulin aggregation. For this analysis, the normalized ThT intensity at 483 nm vs. log [P4 or P5] was plotted.

Figure 1 shows the changes in the ThT fluorescence intensity of insulin solutions at 483 nm in absence and presence of different concentrations of P4 and P5 (from 0 to 750 μM) after incubating at 60°C for 72 h. ThT fluorescence intensity started decreasing as the concentration of peptides was increased until it approached a peptide concentration of 500 μM. Beyond this concentration, peptide inhibitory effects got saturated (at 750 μM). These results indicated that both peptides inhibited the insulin fibrillation, and their effects were dose-dependent. Irrespective of the type of peptides, the maximal inhibitory effect was observed at ≈500 μM. Although both peptides inhibited the aggregation of insulin, the inhibitory effect of P4 and P5 were not similar. Our results revealed that P4 was more effective than P5 in attenuating the insulin amyloid aggregation (cf. Figures 1A,B). Since the maximum inhibition of insulin fibrillation was achieved at 500 μM, in the subsequent experiments we have excluded the higher concentration of peptide; i.e., 750 μM.

**Figure 2:**
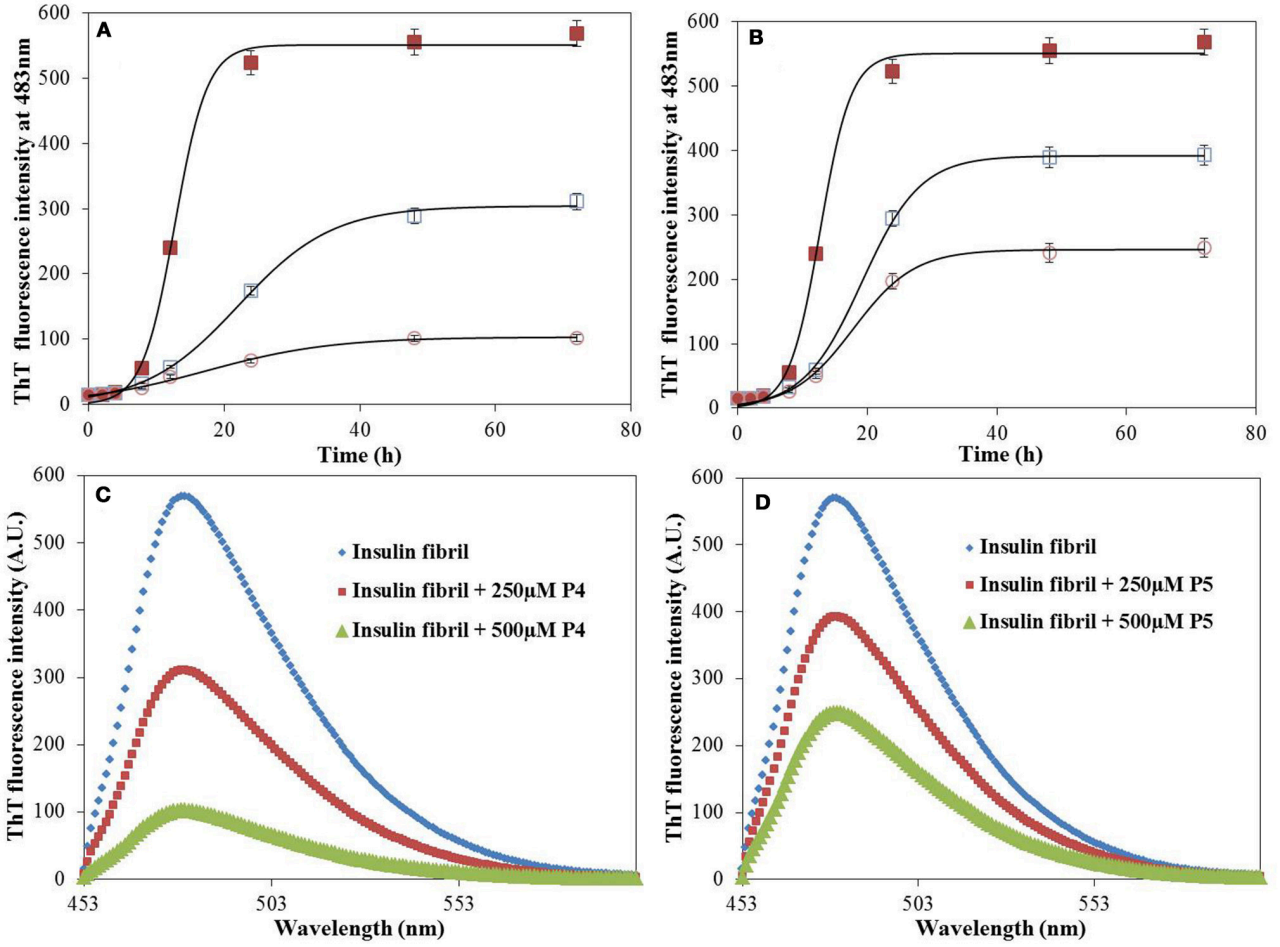
Time course of insulin fibrillation. The time-dependent ThT kinetics of insulin incubated at 60°C for 72 h in the absence (filled square), and presence of P4 **(A)** and P5 **(B)** at concentration of 250 μM (open square), 500 μM (open circle). ThT fluorescence spectra of insulin in the absence and presence of P4 **(C)** and P5 **(D)**. Experimental data represent the average ± s.d.

Figures 2A,B shows the time course of the insulin fibrillation in absence and presence of P4 and P5. Insulin alone exhibited typical sigmoidal fibrillation kinetics curve with a lag-phase, suggesting the nucleation dependent polymerization. Note that similar behavior was also observed by Wang et al. (2011). The kinetic curve possesses a lag-phase of 5 h, after which fluorescence started to increase in an exponential manner and achieved the stationary phase at 24 h. Our results showed that P4 decreased the final fluorescence intensity by a factor of ≈5.6 and increased the time to attain the plateau. This indicates that P4 executed its anti-amyloidgenic effect by altering the rate of fibril formation of insulin (Botz et al., 2015). In contrary, P5 decreased the final fluorescence intensity by a factor of ≈2.5 (almost half as compared with P4) and had insignificant effect on the time to attain the plateau phase. This suggested the involvement of different inhibition mechanisms for P4 and P5. Botz et al. also observed the similar behavior during amyloid inhibition by short peptides (Botz et al., 2015). Further, we also calculated the effect of P4 and P5 in terms of percent inhibition and found that P4 and P5 were 82 and 56% effective respectively in preventing aggregation (Supplementary Figure S2). The reason behind the difference in the inhibition mechanisms of peptides might be their short length and the presence of aromatic residue, which provide an easy way to find and get settled between the partially unfolded structures formed at the amyloidogenic conditions. Figures 2C,D shows the ThT spectra measured for solutions containing insulin fibrils as well as for insulin co-incubated with P4 and P5 at 60°C for 72 h. Control experiments performed in the absence of insulin showed negligible ThT fluorescence in the presence of P4 and P5 peptides, under the same experimental conditions (Supplementary Figure S3), thereby ruling out any interference or artifacts in the ThT fluorescence assay. In order to extend the physiological relevance of this work, inhibitory effect of peptides on amyloid formation was also studied at pH 7.4 and 37°C (Supplementary Figure S4). These results suggest that peptides retard the amyloid formation even at physiological conditions. However, it requires 240 h (Alam et al., 2017) (longer than 72 h at acidic pH) to form amyloids. Overall, the above results demonstrate that our peptides may act as a general inhibitor for the protein aggregation.

**Figure 3:**
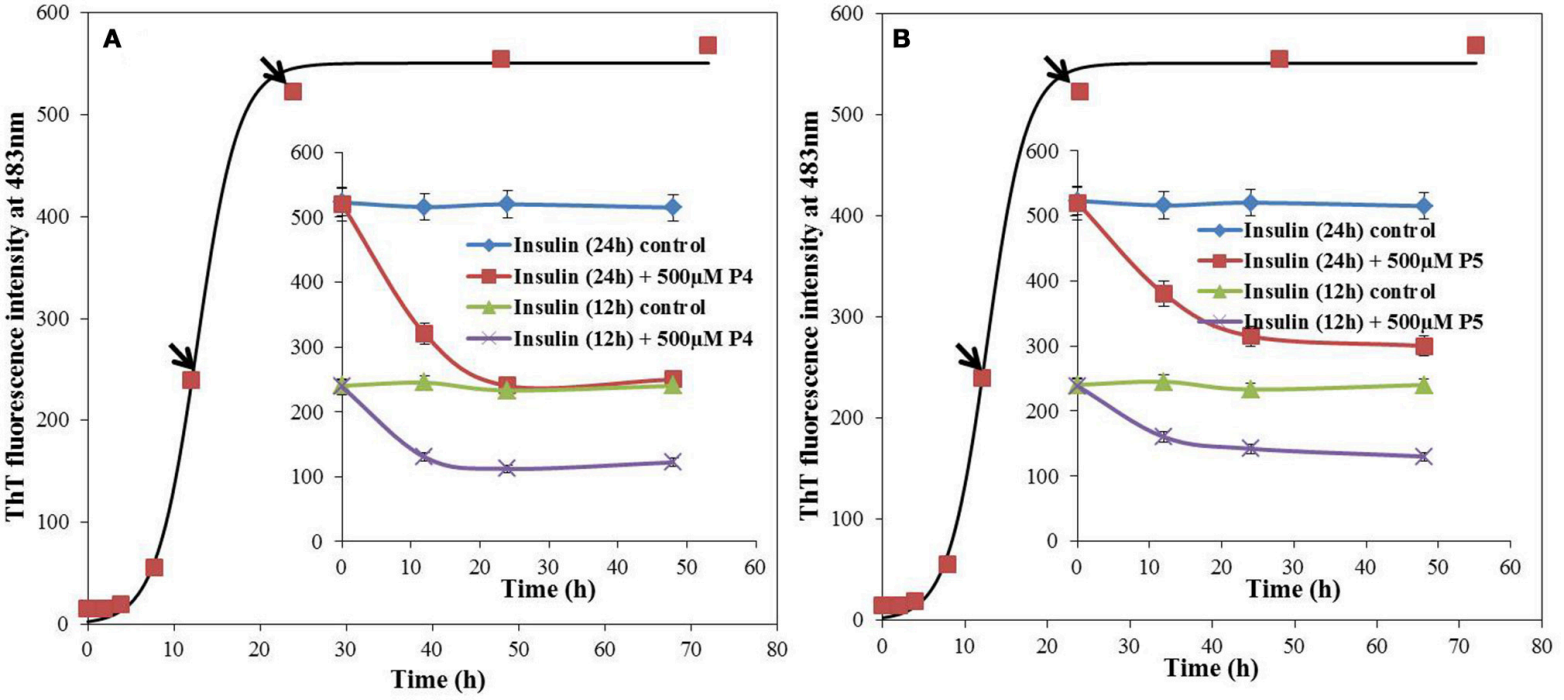
Effect of adding peptide (P4 and P5) at different stages of insulin fibrillation pathway, monitored by ThT fluorescence. 500 μM of P4 **(A)** and P5 **(B)** were added to 12 and 24 h post incubated insulin. Inset, blue and green solid lines represents the ThT fluorescence intensity profiles for insulin in absence of peptides whereas red and violet solid lines represents the post incubated samples with peptides, at 37°C.

The IC_50_ values of P4 and P5 were also evaluated from the dose response curves (Figures 1C,D), which were found to be 278 and 446 μM, respectively. Furthermore, we wanted to know, whether these peptides may block the elongation phase. In order to test this hypothesis, we added the peptides at two different time of incubation (Figure 3). It is clear from data shown in Figure 3 that both peptides arrested the elongation phase of insulin aggregation and also significantly decreased the ThT intensity. These results suggested that P4 and P5 are able to inhibit the amyloid aggregation of insulin and also block the growth of fibrils. From the ThT results we can conclude, (1) pentapeptides with high predisposition to form β-sheet structure inhibit the amyloid aggregation; (2) either premixing of peptides with insulin or the addition of peptide during the fibril growth phase attenuate the amyloid fibril formation.

### Surface hydrophobicity modulation of insulin by peptides

**Figure 4:**
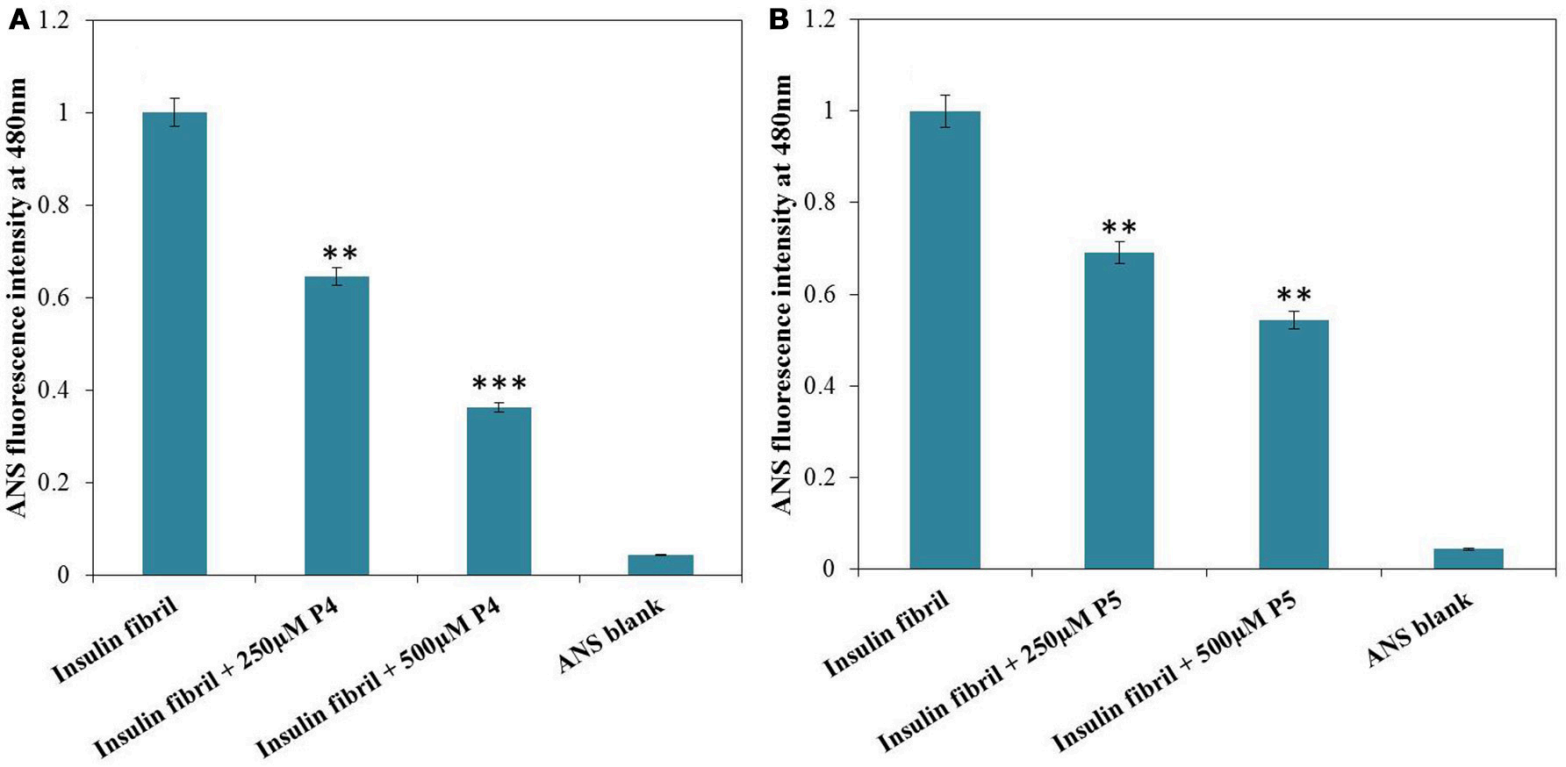
ANS fluorescence of insulin incubated at 60°C for 72 h in absence and presence 250 and 500 μM of P4 **(A)** and P5 **(B)**. ^**^P < 0.01 and ^***^P < 0.001 compared with insulin fibril.

Protein (at least partial) unfolding and subsequent misfolding are believed to be prerequisite steps for the aggregation of proteins. Since core of a globular protein is enriched in hydrophobic amino acid residues as compared to the surface, the protein unfolding and/or misfolding results in exposure of these hydrophobic residues to the external environment. Such alteration of protein structure can be detected by characteristic changes in ANS fluorescence (Dobson, 2003; Younan and Viles, 2015). ANS is an aromatic, hydrophobic, charged fluorescent probe that is very useful to follow conformational changes of proteins in solution. In application to protein unfolding/misfolding, this dye enables detection of the formation and disruption of solvent-exposed hydrophobic patches. The preferential binding of ANS to hydrophobic clusters gives rise to an enhancement in fluorescence emission accompanied by a blue-shift of the spectral maximum (Messa et al., 2014; Siddiqi et al., 2017). ANS showed insignificant fluorescence upon interaction with native insulin (data not shown). Figure 4 depicts the ANS fluorescence spectra measured for insulin samples incubated without and with peptides. Insulin fibrils exhibited strong ANS fluorescence, whereas in the presence of peptides, low ANS intensity was observed. Furthermore, decrease in fluorescence intensity was more pronounced for solutions containing insulin with P4 as compared to insulin solution with P5. These results suggested that peptides probably altered insulin structure masking the available hydrophobic patches. This ability to interact with such patches is likely to be related to their inhibitory potential against insulin fibrillation (Alam et al., 2017). Control experiments performed in the absence of insulin showed negligible ANS fluorescence in the presence of P4 and P5 peptides, under the same experimental conditions (Supplementary Figure S3).

### Acrylamide quenching assay

**Figure 5:**
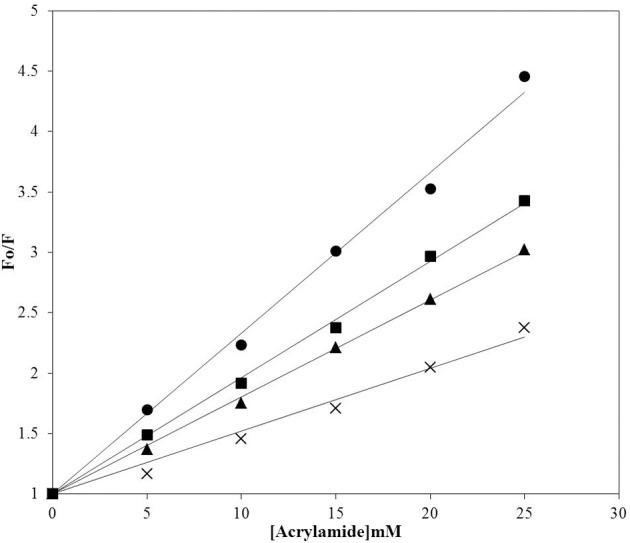
Acrylamide quenching of the intrinsic fluorescence of insulin. Quenching of insulin fluorescence was analyzed in samples incubated at 60°C for 72 h in the absence and presence of P4 and P5. Insulin at 25°C (circles); insulin fibril (crosses); insulin incubated with P4 (squares), insulin incubated with P5 (triangles). F_o_ and F are the fluorescence intensity in absence and presence of acrylamide respectively.

Intrinsic fluorescence can be used to monitor changes in protein conformation. Insulin does not have tryptophan residues, but has four tyrosine residue that can be used as intrinsic fluorophores, Tyr-A^14^, Tyr-A^19^, Tyr-B^16^, and Tyr-B^26^, which exhibit emission upon excitation at 276 nm. The changes of the emission at 302 nm can therefore reflect the changes in the local environment of all four tyrosine residues (Ahmad et al., 2004; Bekard and Dunstan, 2009). However, fluorescence quenching of Tyr residues by acrylamide has been often used as a tool to characterize the solvent accessibility of these amino acid residues in proteins and the polarity of their local environments (Eftink and Ghiron, 1976; Strambini and Gonnelli, 2010). Since acrylamide is a small and neutral compound able to quench the fluorescence of exposed and buried Trp residues principally via collisional mechanism (Eftink and Ghiron, 1976; Strambini and Gonnelli, 2010), the state of the tyrosine solvent accessibility in insulin at different conditions was analyzed by conducting the dynamic fluorescence quenching experiments using acrylamide. Native insulin was more readily quenched by acrylamide than the insulin fibrils (Figure 5), suggesting that fibrillations make tyrosines less accessible to solvent. Acrylamide, being a hydrophilic dye, cannot penetrate to the tightly packed hydrophobic core of insulin fibrils and therefore shows less efficient quenching compared to the effect on monomeric proteins (Chapman et al., 2003). Importantly, noticeably lower quenching levels were also observed for insulin in presence of peptides. This suggests that fibrillation of insulin and interaction of this protein with peptides generated different local environments around its tyrosine residues (Librizzi et al., 2014).

### Effect of peptides on secondary structure during insulin fibrillation

**Figure 6:**
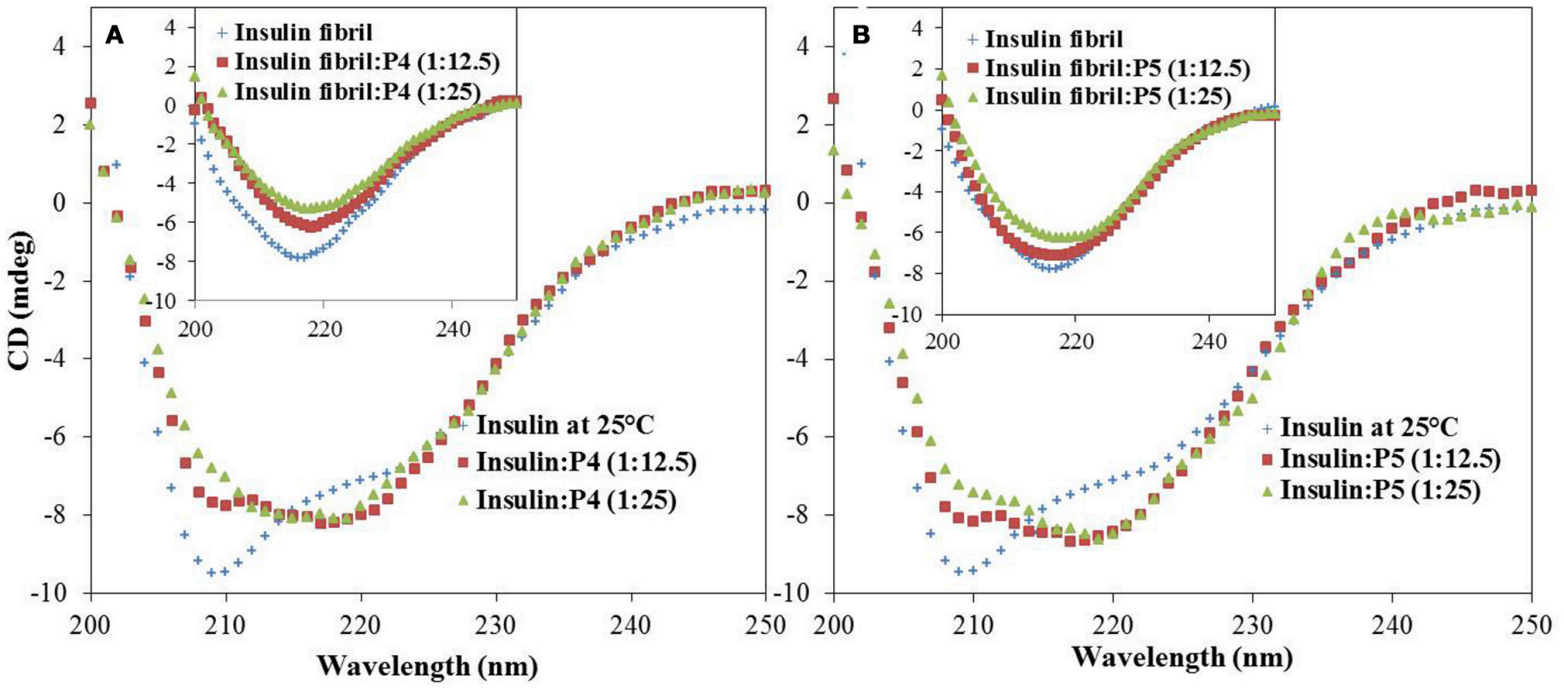
Effect of peptides on insulin secondary structure during fibrillation. Far-UV CD spectra of insulin in the absence and presence of P4 and P5; 250 μM **(A)** and 500 μM **(B)** at 25°C. Inset shows the corresponding far-UV CD spectra of insulin fibrils and insulin co-incubated with P4 and P5 at 60°C for 72 h.

To determine the initial insulin secondary as well as tertiary structure and to monitor the conformational changes in insulin induced by interaction with peptides or caused by fibril formation, far and near-UV CD spectroscopy was utilized, respectively. Far-UV CD is a widely used technique for the determination of alterations in the secondary structure of a protein (Li et al., 2015; Alam P. et al., 2016). In agreement with previous reports (Ahmad et al., 2004; Alam et al., 2017), the far-UV CD spectrum of insulin alone exhibited two negative minima at 222 nm and 208 nm, characteristic of α-helical proteins (Figure 6). The changes in protein secondary structure upon incubation of insulin with peptides (P4 and P5) at 25°C were rather insignificant as it follows from little changes in the intensity and shape of the CD spectra. However, upon incubation of insulin at 60°C for 72 h, far-UV CD spectra underwent dramatic changes. In fact, minima at 208 and 222 nm were lost and a new minima appeared at around 218 nm, suggesting the formation of β-sheet rich structures (Siddiqi et al., 2018). Strikingly, upon incubation with peptides, the shape of the insulin far-UV CD spectra was still indicative of β-sheet rich structure, but the intensity at 218 nm was decreased. These results suggested that peptides inhibited the amyloid aggregation by decreasing the formation of β-sheet rich structures, instead of stabilizing the native α-helical state. Moreover, to make it more informative, we also record the CD spectra of peptides (P4 and P5) alone under different experimental conditions, results inferred a beta sheet like pattern (Supplementary Figure S5).

### Dynamic light scattering measurements

**Figure 7:**
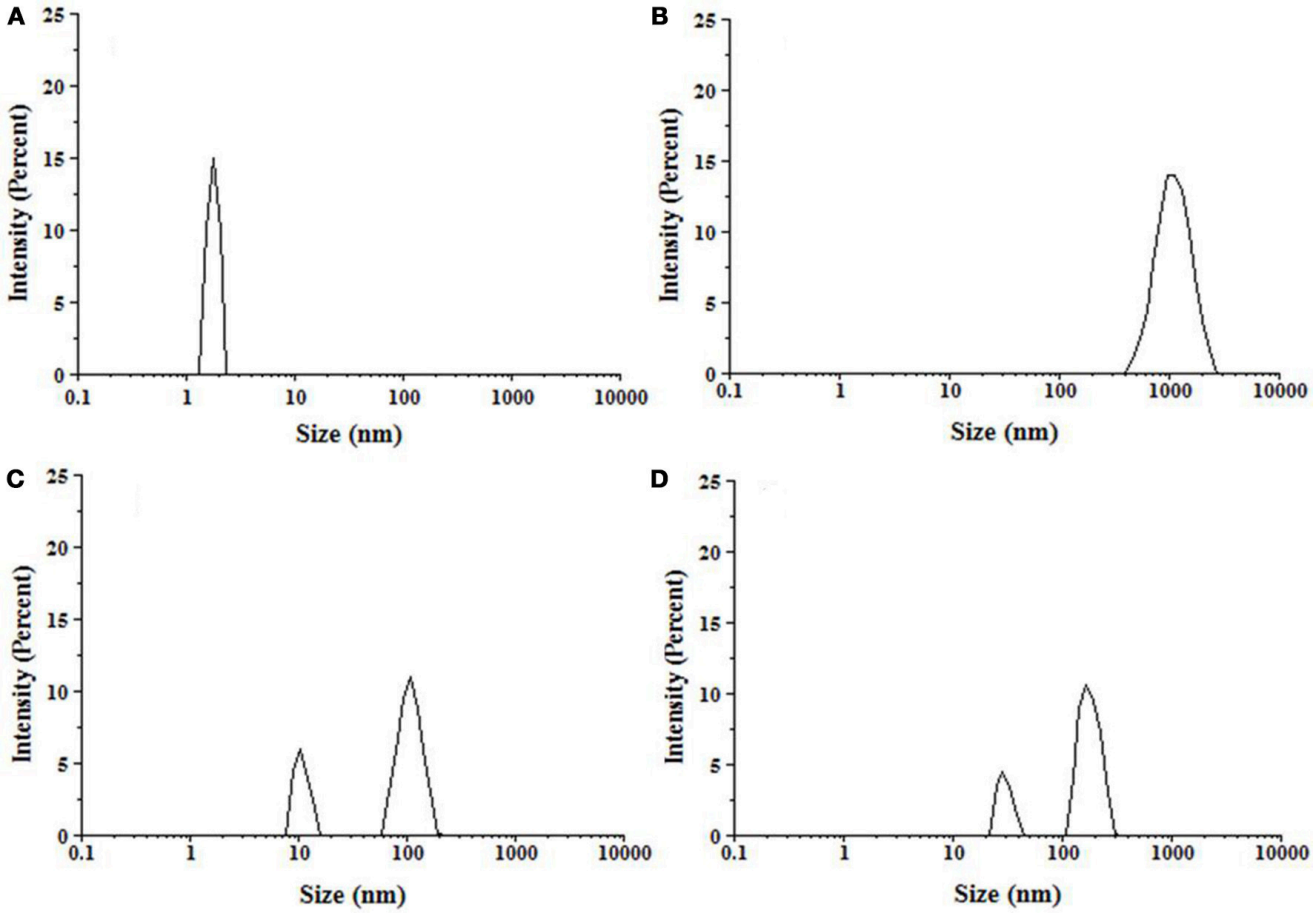
Particle size distribution of insulin samples measured by DLS. **(A)** Insulin at 25°C, **(B)** Insulin incubated at 60°C for 72 h, **(C)** Insulin + 500 μM P4 at 60°C for 72 h, **(D)** Insulin + 500 μM P5 at 60°C for 72 h.

The technique of dynamic light scattering (DLS) is widely employed for defining the hydrodynamic dimensions of particles in liquid phase (Banerjee et al., 2013; Gong et al., 2014). Here, we have monitored the change in the hydrodynamic radii (*R*_*h*_) of insulin during fibrillation in the absence and presence of peptides by DLS. Figure 7 represents the size distribution of insulin in absence and presence of peptides, with the results being plotted as scattered intensity vs. particle size. Insulin alone showed the *R*_*h*_ value of 2 nm, whereas upon incubation for 72 h, formation of large aggregates of about 1,000 nm was observed. These observations are in accordance with the previous reports (Sneideris et al., 2015; Zhu et al., 2016). However, samples of insulin co-incubated with P4 showed two populations of particle with the hydrodynamic radii of 10.1 and 106 nm, which were significantly lower than dimensions found when insulin was incubated alone. In presence of P5, particle with the radii of 28.2 and 164 nm were observed. These results indicated that both peptides were able to interfere with the aggregation process and resulted in the formation of smaller size aggregates, with P4 leading to formation of aggregates with comparatively smaller size. These results were in agreement with a report stating that quinones inhibited amyloid aggregation and resulted in the formation of smaller size aggregate (Gong et al., 2014).

### Effect of peptides on fibril morphology

**Figure 8:**
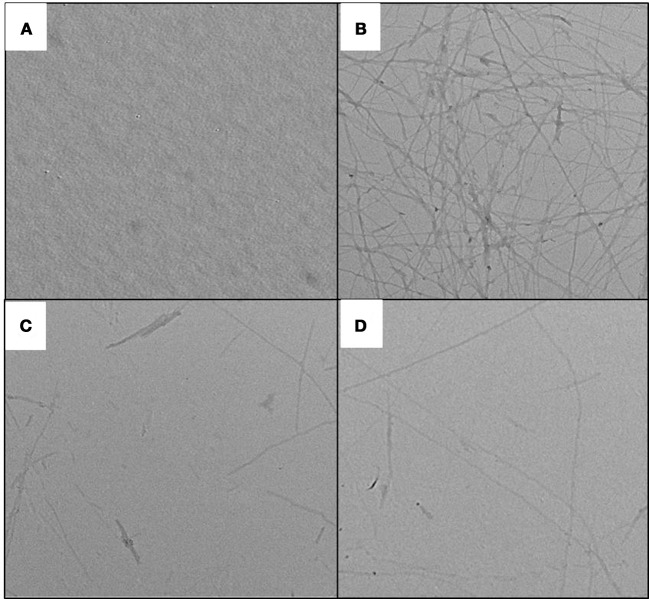
TEM images of insulin assemblies. **(A)** native insulin at 25°C; **(B)** Insulin incubated at 60°C for 72 h in absence of peptides; **(C)** Insulin incubated at 60°C for 72 h in presence of 500 μM P4; and **(D)** Insulin incubated at 60°C for 72 h in presence of 500 μM P5.

Changes in ThT fluorescence intensity of insulin in absence and presence of peptides can be potentially attributed to the change in fibril morphology. This hypothesis was confirmed by TEM (transmission electron microscopy) experiments. TEM is widely used to visualize the amyloid fibrils (Juárez et al., 2009). Insulin at 25°C exhibited no visible fibrils, but upon incubation at 60°C for 72 h, the dense network of amyloid fibril was observed (Figure 8). Morphologically, this sample was characterized by long, straight, and partly bundled fibrils (Whittingham et al., 2002). On the other hand, samples of insulin co-incubated with peptides showed significantly less fibrils. These results supported our ThT results that peptides were able to decrease the ThT fluorescence by restricting the formation of cross β-sheet-rich fibrillar structure. Furthermore, compared to P5, P4 was more effective in reducing the formation of fibrillar structure.

### Effect of peptides on cytotoxicity of aggregated insulin: hemolytic assay

**Figure 9:**
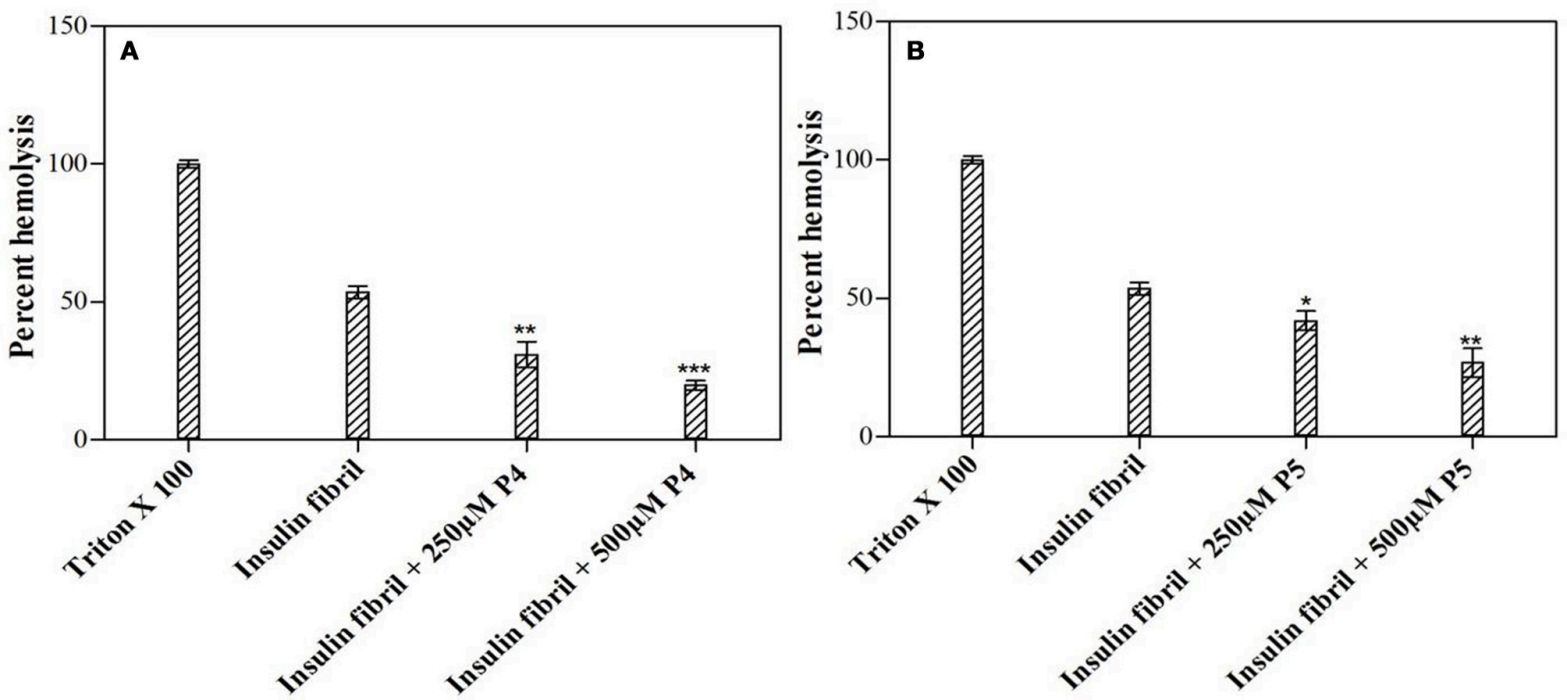
Short peptides attenuated insulin fibril-induced cytotoxicity. Figure shows the inhibitory effects of the P4 **(A)** and P5 **(B)** peptides on insulin fibril-induced hemolysis. Hemolysis of erythrocytes was induced by incubating the cell suspensions with insulin fibrils. ^*^P < 0.05, ^**^P < 0.01, and ^***^P < 0.001 compared with insulin fibril.

In hemolytic assay, we monitored the release of hemoglobin in plasma following red blood cell (RBC) lysis upon exposure to agent under consideration. In this context, some amyloid fibrils have been reported to disintegrate cell membrane and cause cell death (Grudzielanek et al., 2007; Wang et al., 2011). We used these observations to investigate the dampening effect of peptides on amyloid-induced toxicity in human RBCs. Figure 9 represents the results of the hemolytic assays of insulin alone as well as co-incubated with peptides P4 and P5. Results of this analysis indicated that insulin fibrils exhibit strong cell damaging effect, with the hemolysis rate of 53.50%, which was higher than the control. Likely, this observed lytic effect was due to the membrane permeation induced by amyloid fibrils (Mark et al., 1997). On the other hand, co-incubation of insulin with peptides noticeably decreased hemolysis. Furthermore, this cytoprotective potential was different for peptides in study. In fact, in the presence of insulin co-incubated with P4 (500 μM), hemolysis was decreased down to 19.76%, whereas P5 (500 μM) was characterized by the decreased capability to reduce lysis (26.79%). These results suggested that peptides inhibited fibril-induced hemolysis in a concentration dependent manner, probably due to the disruption of fibrillar structure, thereby lowering the cytotoxicity of the fibrils (Wang et al., 2011). On the other hand, control study suggested that peptides themselves had no hemolytic or cytotoxic effect, when tested alone on human RBCs (Supplementary Figure S6).

### Interaction of peptide with insulin: docking analysis

**Figure 10:**
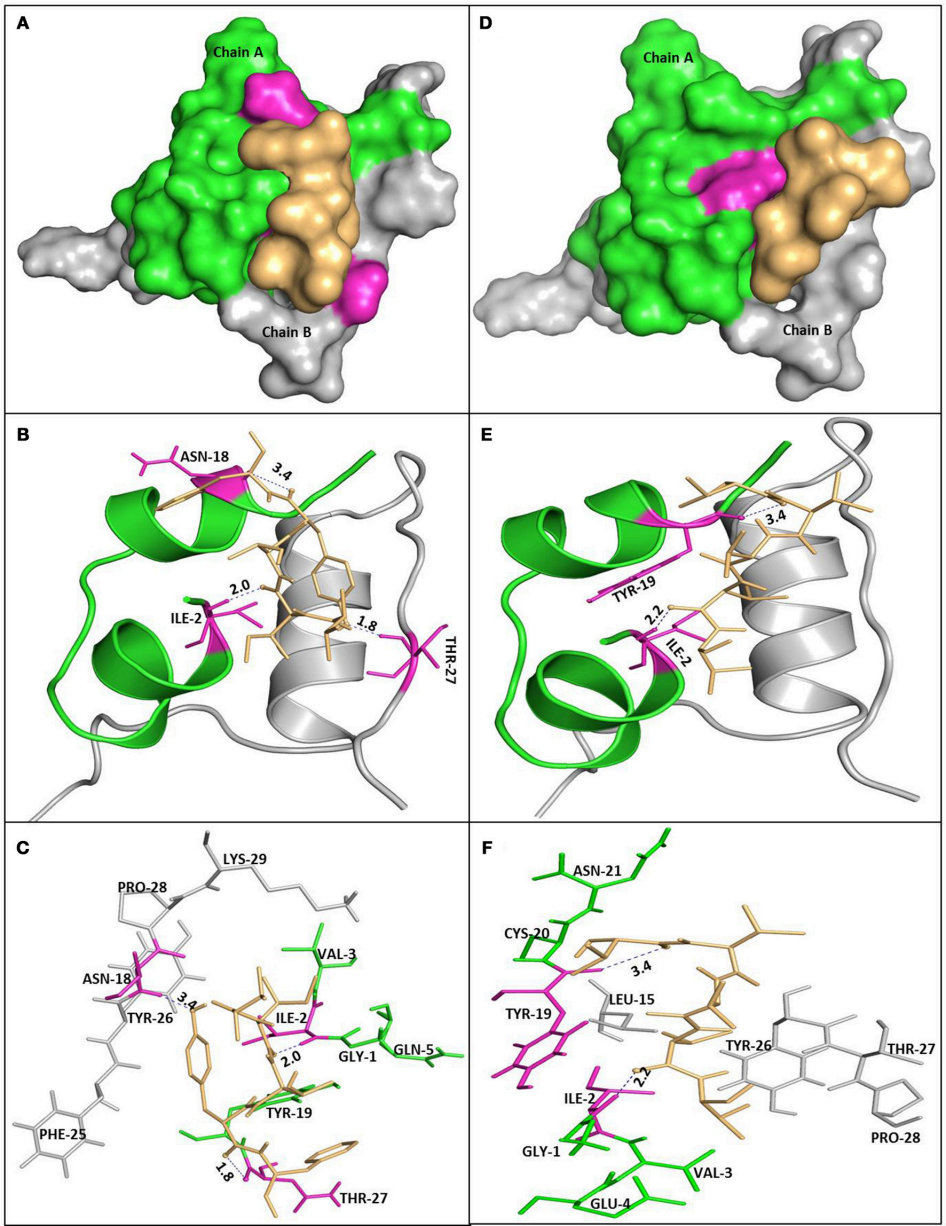
Molecular docking of P4 and P5 with insulin. Molecular surface representation of P4 and P5 docked with insulin (**A,D**, respectively). Peptide is shown in a stick representation, and insulin is represented with ribbon model showing the contact residues (**B,E** for P4 and P5, respectively). Detailed view of the docking poses of P4 **(C)** and P5 **(F)** with insulin.

Molecular docking was used to get structural insights and detailed information on interaction of insulin with the peptides at atomic level. In the present study, Vina Autodock program 4.0 was used to calculate the possible conformation of the peptide that binds to the insulin (Morris et al., 2009). The best energy ranked results are summarized in Table 1 and shown in Figure 10. The free energy for the interaction of P4 and P5 with human insulin were found to be −5.6 and −4.9 kcal M^−1^, respectively. The region responsible for peptide binding seems to include both chain of insulin (A and B). It has been reported that the segment of insulin B chain (^11^LVEALY^16^) is especially prone for amyloid formation. Interestingly, none of the peptides (P4 and P5) interacted with the amyloidogenic insulin region, but they inhibited the insulin amyloid aggregation. This behavior was similar to the nanobody, which inhibited amyloid fibril formation. They reported the effects of two camelid antibody fragments, generally called nanobodies (De Genst et al., 2013; Gong et al., 2014; Alam et al., 2017). Figures 10C,F show the amino acid residues involved in the interaction of insulin with P4 and P5 and that appear to play an important role in inhibiting the insulin amyloid fibril formation. These results suggested that instead of blocking the aggregation prone region, peptides (P4 and P5) inhibited the amyloid formation by restricting the participation of these key residues in the intermolecular interactions during the course of amyloidogenesis (De Genst et al., 2013; Alam et al., 2017).

**Table 1:** Molecular docking parameters of Insulin- peptides interaction.

**Peptide**	**Contact residues**	**Distance (Å)**	**Surrounding residues**	**Common residues of interaction**	**Free energy change (kcal M^−1^)**
P5	**Chain A:** TYR^19^ ILE^2^	3.4 2.2	**Chain A:** GLY^1^, VAL^3^, GLU^4^, ASN^21^, CYS^20^ **Chain B:** LEU^15^, TYR^26^, THR^27^, PRO^2^	**Chain A:** TYR^19^, ILE^2^, VAL^3^	−4.9
P4	**Chain A:** ASN^18^ ILE^2^ **Chain B:** THR^27^	3.4 2.0 1.8	**Chain A:** GLY^1^, VAL^3^, GLN^5^, TYR^19^ **Chain B:** PHE^25^, TYR^26^, PRO^28^, LYS^29^	**Chain B:** PRO^28^, THR^27^, TYR^26^	−5.6

### Effect of peptides on mature insulin fibrils

**Figure 11:**
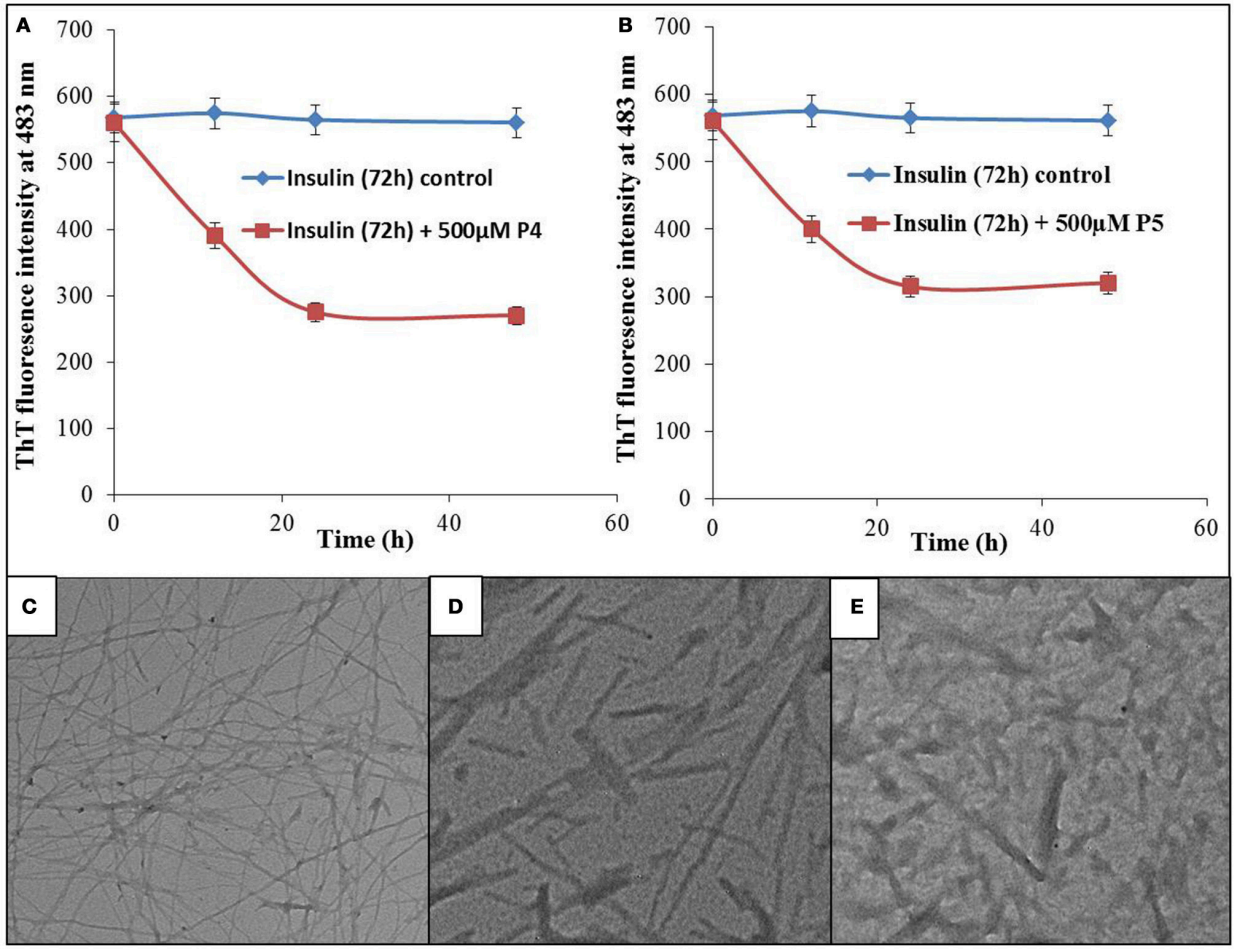
Effect of peptides on preformed fibrils. ThT fluorescence intensity of insulin fibrils and fibrils co-incubated with 500 μM of P4 **(A)** and P5 **(B)** for different time intervals. TEM image of insulin fibrils **(C)**, TEM image of insulin fibrils co-incubated with 500 μM of P4 **(D)** and P5 **(E)** for 48 h.

**Figure 12:**
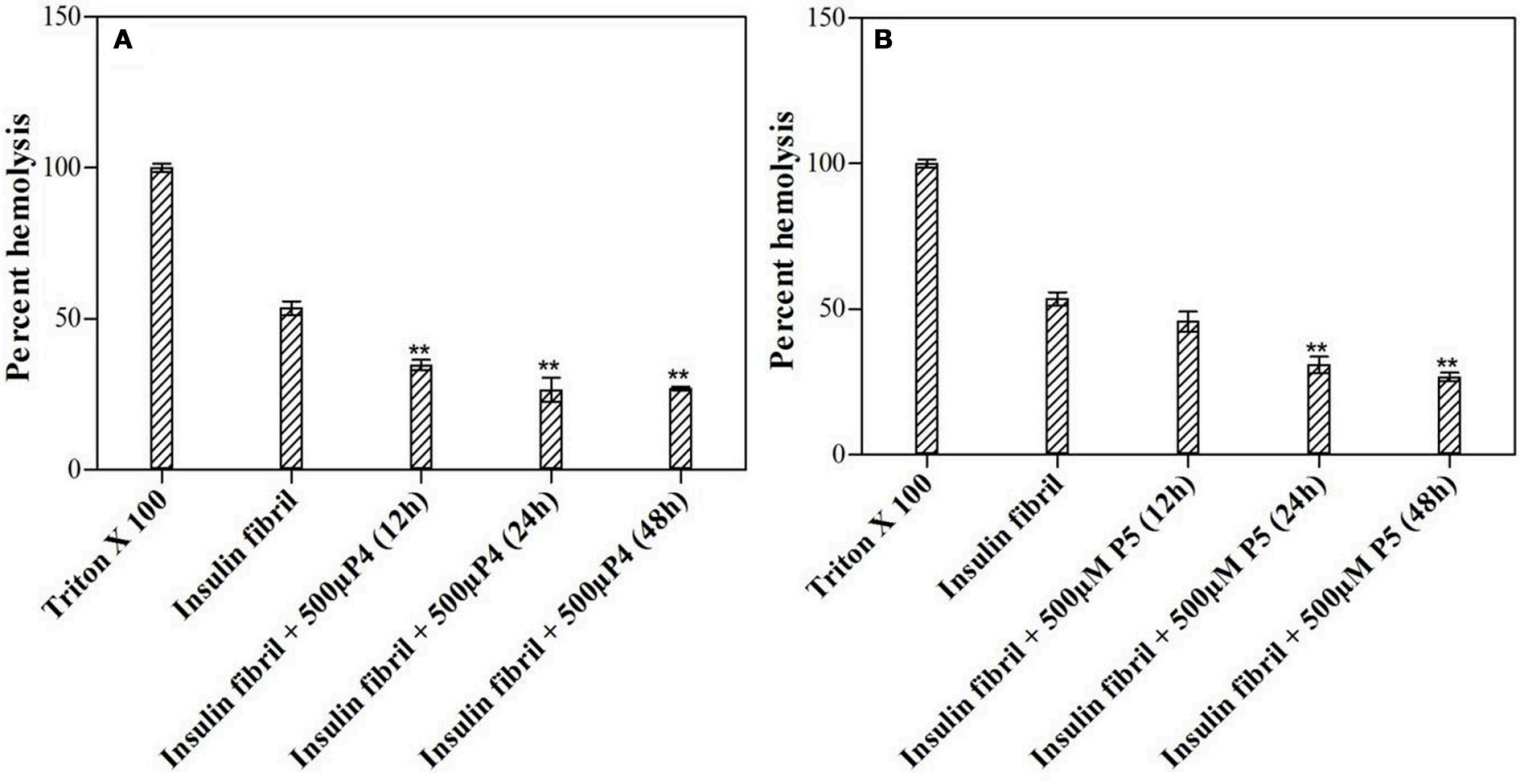
Effect of peptides on preformed fibrils. Hemolytic assay after being exposed to preformed insulin fibrils incubated with or without 500 μM of P4 **(A)** and P5 **(B)**. ^**^P < 0.01 compared with insulin fibril.

Results summarized in this study show that selected peptides possess the potential to inhibit the insulin fibrillation and thereby protect against amyloid-induced cytotoxicity. Then, we tested our next hypothesis that in addition to their fibrillation inhibitory potential, these peptides may also possess the ability to disaggregate the preformed fibrils. To validate this hypothesis, we conducted the ThT fluorescence assay of insulin fibril in the absence and presence of peptides (see Figures 11A,B). When insulin fibrils were co-incubated with the peptides, ThT fluorescence intensity started to decrease over time and eventually attained a saturation level, suggesting that peptides do possess fibril disaggregating ability. Strikingly, when compared with P4, effects obtained with P5 were not significantly different. To better understand the effect of treatment with peptides on fibril morphology, we performed the TEM analysis. The obtained images showed that following the incubation of preformed fibril with peptides, the fibrillar structure was significantly altered when compared with the insulin fibril alone (Figures 11C–E). These results suggested that peptides possessed the fibril disaggregating potential. Similar behavior was observed when preformed fibrils were incubated with morin hydrate (Noor et al., 2012) and silibinin (Young et al., 2015). Furthermore, we also checked the effect of samples of peptide-treated fibrils on the lysis of RBCs and found that samples co-incubated with peptides possessed significantly reduced RBC lysis potential (Figure 12).

## Conclusions

In this work, we presented evidence that short peptides (namely P4 and P5) despite having inherent predisposition to form β-sheet structure, are able to inhibit the formation of cross β-sheet rich structure during insulin fibrillation. This inhibitory potential was shown by the ThT, DLS, and TEM analyses. Although both P4 and P5 possessed the potential to inhibit amyloidogenesis, P4 exerted higher effect, probably due to the presence of the aromatic amino acid residues, such as Tyr and Phe. This peptide has little effect on the lag-phase but greatly altered the time required to attain the plateau and remarkably reduced the extent of fibrillation. Our data also showed that pentapeptides, besides inhibiting amyloid formation, is also able to restrict the elongation process and disaggregate the preformed mature fibrils. Furthermore, CD analysis suggested that although the peptides were unable to stabilize the native α-helical structure of insulin during the course of its aggregation, they exerted their anti-amyloidogenic behavior by reducing the formation β-sheet-rich structure. Therefore, in this study, we explored the mechanism by which short peptides are able to significantly inhibit the *in vitro* aggregation of insulin and dramatically reduce the hemolytic effect of insulin fibrils. Overall our data suggest that these short peptides offers a new strategy for designing of novel peptide-based inhibitors of pathological protein aggregation.

## Author contributions

MS, PA, VU, and RK: designed the studies and analyzed the data; MS, PA, TI, NM, AA, MA, and SM: undertook the experimental work; MS, PA, SN, VU, MA, and RK: contributed to figure and manuscript preparation.

### Conflict of interest statement

The authors declare that the research was conducted in the absence of any commercial or financial relationships that could be construed as a potential conflict of interest.
