# Supplementary material for: Elucidating the Inhibitory Potential of Designed Peptides Against Amyloid Fibrillation and Amyloid Associated Cytotoxicity
Source: Front Chem. 2018 Aug 3;6:311. doi: 10.3389/fchem.2018.00311 (PMC6085999; doi:10.3389/fchem.2018.00311)
Supplement: Supplementary file 1 [file Data_Sheet_1.docx]

**Supplementary information**

**Elucidating the inhibitory potential of designed peptides against amyloid fibrillation and amyloid associated cytotoxicity**

Mohammad Khursheed Siddiqi^1^, Parvez Alam^1^, Tabish Iqbal^1^, Nabeela Majid^1^, Sadia Malik^1^, Saima Nusrat^1^, Aftab Alam^2^, Mohd Rehan Ajmal^1^, Vladimir N. Uversky^3,4,5^, Rizwan Hasan Khan^1,^*

^1^Interdisciplinary Biotechnology Unit, Aligarh Muslim University, Aligarh-202002, India

^2^ Center for Interdisciplinary Research in Basic Science, Jamia Millia Islamia, New Delhi, India- 110025

^3^Protein Research Group, Institute for Biological Instrumentation of the Russian Academy of Sciences, Institutskaya Str., 7, Pushchino, Moscow region, Russia, 142290;

^4^Department of Biological Sciences, Faculty of Sciences, King Abdulaziz University, P.O. Box 80203, Jeddah, Saudi Arabia;

^5^Department of Molecular Medicine and USF Health Byrd Alzheimer's Research Institute, Morsani College of Medicine, University of South Florida, Tampa, FL, USA

***To whom correspondence should be addressed**: Prof. Rizwan Hasan Khan, PhD, Interdisciplinary Biotechnology Unit, Aligarh Muslim University, Aligarh-202002, U.P., India. **E-mail:** [rizwanhkhan1@gmail.com](mailto:rizwanhkhan1@gmail.com); Phone: +91-571-2720388; Fax: + 91-571-2721776


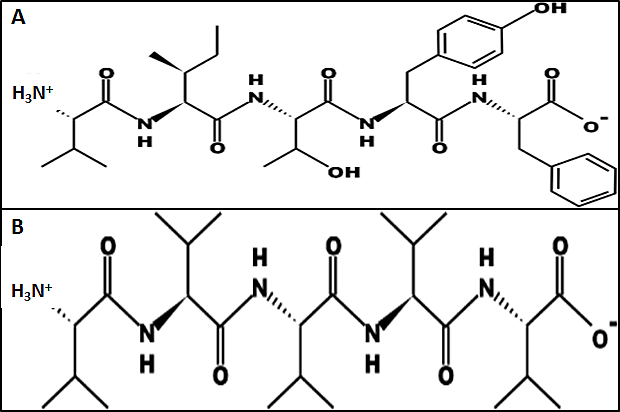


**Supplementary Figure S1:** Primary structure of P4 (A) and P5 (B) using Pepdraw.


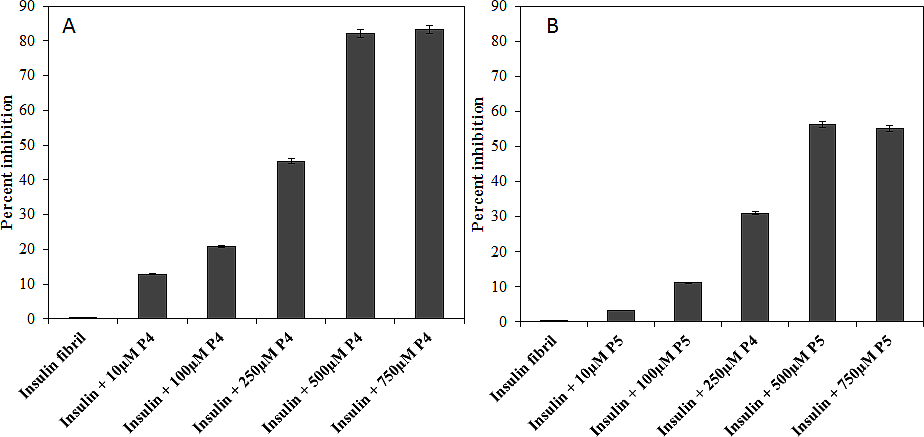


**Supplementary Figure S2:** Percentage ThT fluorescence inhibition of insulin when incubated at 60^o^C for 72 h in absence and presence of P4 (A) and P5 (B).


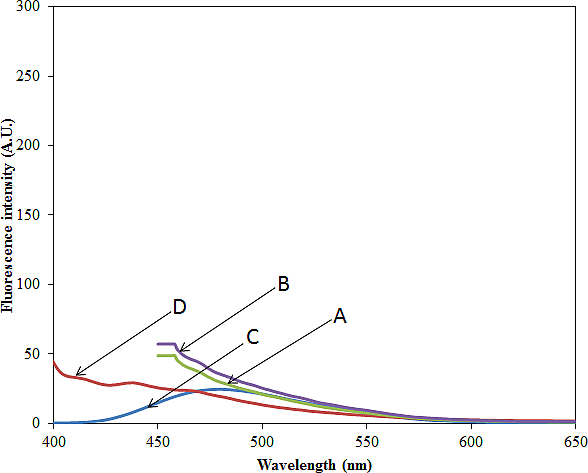


**Supplementary Figure S3:** ThT fluorescence spectra of P4 and P5 peptide only (A and B respectively) at 60^o^C for 72 h. ANS fluorescence spectra of P4 and P5 peptide only (C and D respectively) at 60^o^C for 72 h.


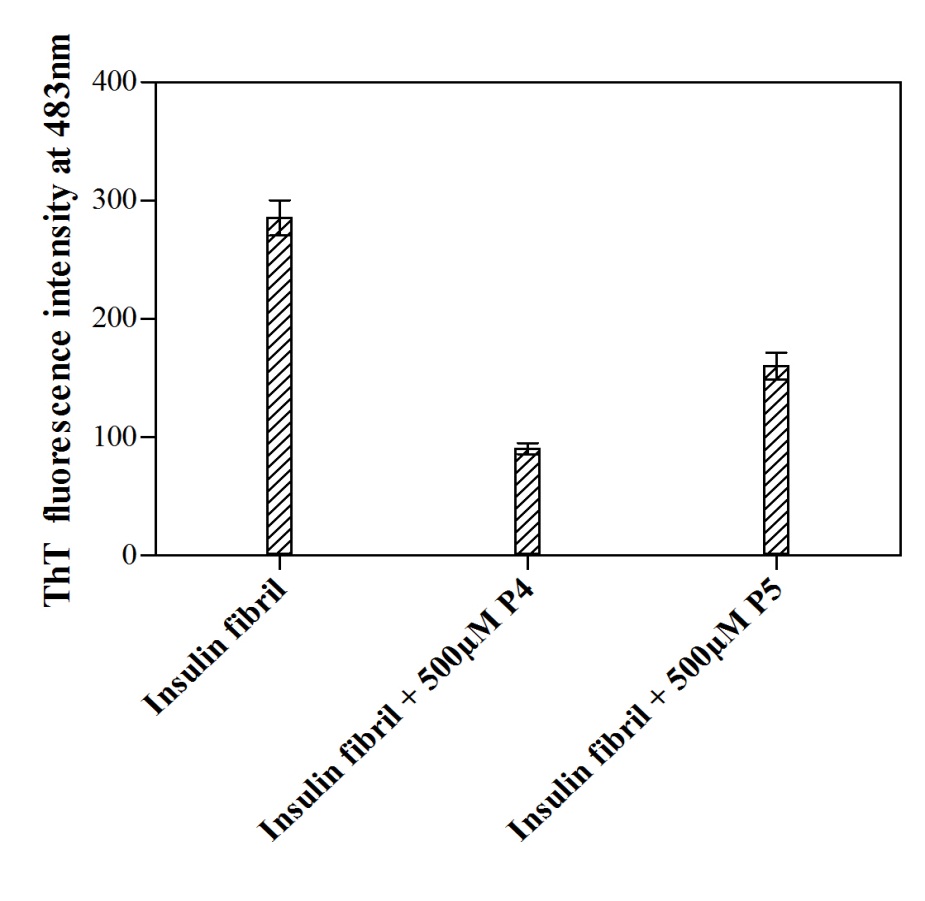


**Supplementary Figure S4:** ThT fluorescence intensity of insulin in absence and presence of P4 (500 µM) and P5 (500 µM). Samples were incubated at 37 °C and pH 7.4 for 240 hours.


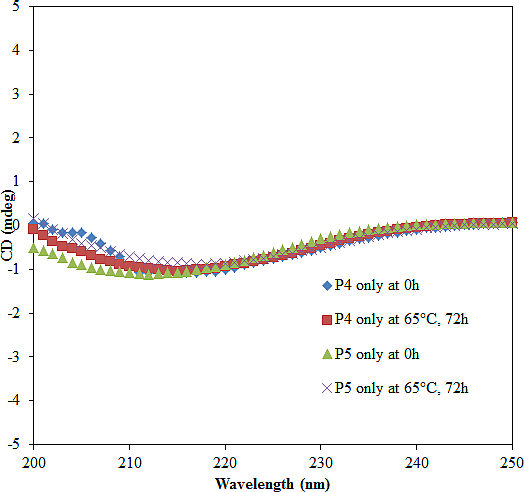


**Supplementary Figure S5:** Far UV CD spectra of P4 and P5 peptides only at different point of incubation. Concentration of each peptide was taken as 500µM.


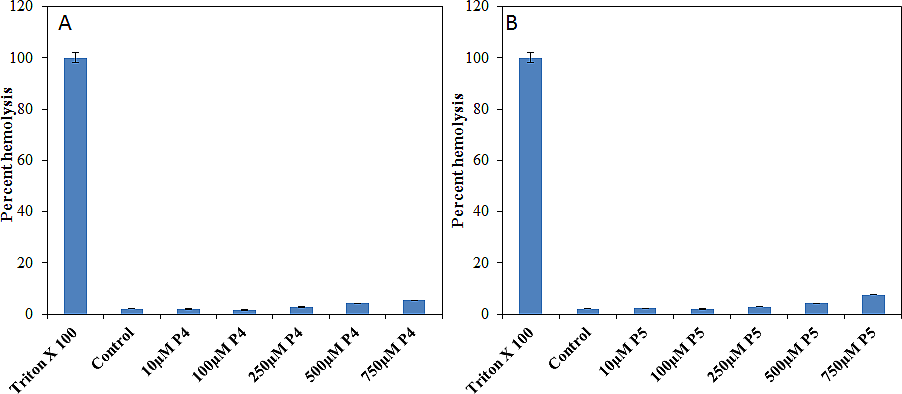


**Supplementary Figure S6:** Percentage hemolysis of P4 (A) and P5 (B) on human red blood cells for different concentrations (0-750 µM) of peptides. Experimental data represent the average ± s.d.
